# Prevalence of medication overuse headache in an interdisciplinary pain clinic

**DOI:** 10.1186/1129-2377-14-4

**Published:** 2013-01-30

**Authors:** Corinne Wanner Schmid, Konrad Maurer, Daniel M Schmid, Eli Alon, Donat R Spahn, Andreas R Gantenbein, Peter S Sandor

**Affiliations:** 1 Institute of Anesthesiology, University Hospital of Zurich, Rämistrasse 100, Zurich 8091, Switzerland; 2 Institute of Physiology and Zurich Center for Integrative Human Physiology (ZIHP), University of Zurich, Zurich, Switzerland; 3 Department of Urology, University Hospital Zurich, Zurich, Switzerland; 4 Neurorehabilitation, RehaClinic Bad, Zurzach/Baden, Switzerland

**Keywords:** Medication overuse, Headache, Interdisciplinary pain management, Chronic pain

## Abstract

**Background:**

Medication overuse headache (MOH) has been recognized as an important problem in headache patients although the pathophysiological mechanisms remain unclear. The diagnosis of MOH is based on clinical characteristics defined by the International Headache Society. The aim was the evaluation of the diagnostic criteria of MOH in a mixed population of chronic pain patients to gain information about the prevalence and possible associations with MOH.

**Methods:**

Data of all patients referred to the interdisciplinary pain clinic at the University Hospital of Zurich between September 2005 and December 2007 were retrospectively analyzed. Demographic data (age, sex, history of migration), as well as data about duration of pain disease, category of pain disease (neurological, psychiatric, rheumatologic, other), use of medication, history of trauma, and comorbidity of depression and anxiety have been collected.

**Results:**

Totally 178 of 187 consecutive chronic pain patients were included in the study. A total of 138 patients (78%) used analgesics on 15 or more days per month. Chronic headache was more prevalent among patients with analgesic overuse (39.8%) than without analgesic overuse (18%). The prevalence of MOH was 29%. The odds ratio (OR) for a patient with medication overuse to have chronic headache was 13.1 if he had a history of primary headache, compared to a patient without a primary headache syndrome. Furthermore, history of headache (OR 2.5, CI [1.13;5.44]), history of migration (OR 2.9, CI [1.31;6.32]) and comorbid depression (OR 3.5, CI [1.46;8.52]) were associated with overuse of acute medication, in general.

**Conclusions:**

Primary headaches have a high risk for chronification in patients overusing analgesics for other pain disorders. Whereas history of headache, history of migration and comorbidity of depression are independentely associated with analgesic overuse in this group of patients.

## Background

Medication overuse headache (MOH) is considered to be an important problem among headache patients worldwide with an estimated prevalence of 1–1.5% in the general population [1-5]. Studies mainly performed in tertiary care headache centers found that migraineurs and, to a lesser extent, patients with tension type headache are at risk for worsening of their headache under regular intake of acute relief medication [6]. Although MOH is prevalent in patients of all age groups, a considerable part of the working population is affected. MOH therefore might have an important socioeconomical impact.

MOH is a concept based on clinical observations first described in the late 1950s [7]. The underlying mechanisms are still unclear [8]. Recent findings suggest that the regular intake of an analgesic substance might lead to changes in different neurobiochemical systems; e.g. the serotoninergic system is modulated by the intake of different classes of analgesics which will lead to alterations of the antinociceptive serotoninergic pathway and central sensitization [9-16]. Other mechanisms proposed involve the NMDA-receptor [17], the opioid- system [18], and alterations in membrane transduction [19].

New diagnostic criteria vor MOH were introduced in 2006 [20] (see Table 1 for diagnostic criteria). Population-based studies have identified possible risk factors for the development of chronic headache, such as age, female gender, low socioeconomic status, non-married civil status, obesity, snoring, comorbid musculosceletal pain, head and neck injury, and stressful life events [21,22]. This has raised the question whether chronic headache is the consequence or the cause of overused analgesics [21,23]. The development of “de novo” MOH in patients overusing analgesics would establish this causative relationship, however, prospective studies on that topic are missing. Patients taking analgesics for chronic pain - other than headache - represent an interesting population to examine such a context. Previous studies, using other than the ICHD-II-Appendix criteria for the definition of MOH, showed conflicting results regarding new onset headache under medication overuse [24-28].

**Table 1:** **Overview of the different criteria for Medication overuse headache** (**MOH**)

	**ICHD-I**	**ICHD-II**	**ICHD-II revised**	**ICHD-II Appendix**
	***IHS 1988***	***IHS 2004***	***Silberstein et al****.****2005***	***Olesen et al****.****2006***
Comment	not defined	Different entities for different substances	Elimination of headache characteristics	Elimination of criterion D
**A**		Headache^1^ > 15 days/month	Headache ≥ 15 days/month	Headache ≥ 15 days/month
**B**		Substance overuse ≥ 10/15^2^ days/month	Substance overuse ≥ 10/15^2^ days/month	Substance overuse ≥ 10/15^2^ days/month
		for ≥ 3 months	for > 3 months	for > 3 months
**C**		Headache developed or worsened	Headache developed or worsened	Headache developed or worsened
**D**		Resolution within 2 months after withdrawal	Resolution within 2 months after withdrawal	n.a.

^1^ Headache characteristics defined for different substances.

^2^ ≥ 10 days/month for ergotamines, triptans, opioids or combination analgesics; ≥ 15 days/month for simple analgesics (or combination of any, ICHD-II Appendix).

The objective of the present study was to examine the prevalence of MOH as defined in the new ICHD-II-Appendix criteria in a population of patients with chronic pain different than headache. Our hypothesis was that chronic headache would be more prevalent in patients with analgesic overuse (AO), especially when additionaly suffering from primary headaches, such as migraine or tension type headache.

## Methods

The interdisciplinary pain clinic at the University Hospital of Zurich is a tertiary referral center for chronic pain patients. Participating specialists are an anesthesiologist, a neurologist, a psychiatrist and a rheumatologist. Patients were referred directly by General Practitioners and Specialists from the whole region of Zurich without selection bias from any of the participating specialists. Upon referral, every patient is examined by each specialist separately in the course of one week. The examination consists of a careful and pain-specific history taking and a physical examination. Headache diagnosis of all affected patients are made by a well trained neurologist (PS) according to ICHD-II and ICHD-II-Appendix criteria regarding MOH, respectively [20,29]. At the end of the week patients are dicussed in a meeting to elaborate a comprehensive diagnostic worksheet and to provide a treatment plan, based on clinical findings and pathophysiological considerations. After informing the patient about the results, a comprehensive report is written to the referring general practicioner or specialist.

The study was approved by the local ethical committee and conducted according to the principles of the Declaration of Helsinki. All patients had signed an informed consent to allow subequent use of their data for study purposes. For the study period (September 2005 to December 2007) data were collected from the comprehensive reports retrospectively. If necessary, additional demographic data, information about pain symptoms, pain scores, mood disturbances and drug intake, or specialists’ examination protocols was taken from the patients charts.

### Data collection

Data of all patients referred to the interdisciplinary pain clinic were screened. Exclusion criteria were: headache as the sole pain symptom; incomplete examination (defined as examination by less than 3 specialists) and missing data on medication intake. The information collected for this study included demographic data regarding age; gender; history of migration (defined as having a mother tongue other than one of the four Swiss national languages: Swiss-German, French, Italian, Rumantsch); duration (years) of pain suffering; history of head and/or neck trauma, and use and type of acute medication. The disorders were categorized into: “neurologic”, “rheumatologic”, “psychiatric” and “other”. Headache diagnosis and comorbidity of “depression” and “anxiety” were additionally recorded.

For comparative analysis, patients were categorized in two subgroups “analgesic overuse (AO)” and “no analgesic overuse (noAO)”, in analogy to the criteria for medication overuse headache (MOH) defined by the ICHD-II Appendix (10 or more days for triptans and opioids, 15 or more days for analgesics). Age, gender, years of pain suffering, history of migration, history of headache, history of accident (subsequently refered to as “accident”), depression and anxiety were defined as possible associated factors for analgesic overuse.

### Statistical analysis

For the analysis of the primary hypotheses, Fisher’s exact test comparing binary variables was performed. For the univariate analysis of associations the Fisher-Test was applied for binary and nominable variables. For continous variables, a *t*-test was performed for normally distributed and a Mann–Whitney-U-Test for not normally distributed data. For all significant factors, OR with 95%-confidence interval was provided. A significance level of *p* < 0.05 was defined. For multiple testing Bonferroni-corrected p-values were used. Regarding associations for medication overuse, a multiple logistic regression was performed with all factors significant at univariate analysis, and OR with 95%- confidence interval provided.

## Results

Data of 187 consecutive patients were screened and 178 patients were included in the study. Nine patients were excluded: in seven patients headache was the only pain disease, one patient had an incomplete examination and one patient had incomplete data regarding medication intake. Five patients were not evaluated by the psychiatrist and were therefore excluded from classification of disease categories and analysis of depression and anxiety as associated factors for analgesic overuse.

### Demographic and clinical characteristics of the overall study population

Of the 178 patients, 91 (51%) were female. The mean age was 46 ± 13 years (range 18 to 81 years). A total of 112 (63%) patients had a history of migration. The mean duration of pain suffering was 7.8 ± 9.2 years (range 0 to 50 years) (Table 2). The diagnostic category was rheumatologic in 146 patients, psychiatric in 138, neurologic in 114; and 17 had an “other” diagnostic category. The cumulative number of diagnostic categories per patient was 4 in 3% of the patients, 3 in 48%, 2 in 35% and only one category in 14%. At the time of the assessment 92 patients (52%) reported to have headache, whereas 57 patients (32%) had headache on ≥ 15 days per month. Migraine was the most frequent headache type, diagnosed either as the only headache type or in combination with other headache types in a total of 63 (35%) patients. Tension type headache was found in 16 patients (9%).

**Table 2:** **Demographic data of all the patients**, **as well as the subgroups with and without analgesic overuse**

	**Overall population**	**Analgesic overuse**	**No analgesic overuse**	**p-value**
	**n = 178**	**n = 138**	**n = 40**	
**Age (y, mean** ± **SD)**	46 ± 13	46 ± 12	42 ± 14	ns
**Sex male/ female**	85/ 93	65/ 73	20/ 20	ns
**History of migration (n)**	111	95	16	0.012
**Duration of pain disorder (y)**	7.8 ± 9.2	7.7 ± 8.9	8.0 ± 9.9	ns
**Accident (n)**	53	43	11	ns
**Depression (n)**	82	74	8	0.002
**Anxiety (n)**	22	18	4	ns

### Use of acute medication

Only 28 of 178 (16%) patients did not take any acute medication at all and 12 (7%) patients took analgesics on less than 15 days per month. NSAIDs were the most commonly used drugs (93 patients, 62%), followed by opioids (85 patients, 57%) and paracetamol (77 patients, 51%). Triptans and ergotamins drugs were consumed by 8 (5%) and 1 (1%) patients respectively. Out of the 138 patients overusing analgesics, 131 (95%) had a daily intake. Of them 56 (41%) took one analgesic, 52 (38%) took two different types, 27 (20%) three different types and 3 (2%) patients four different types on analgesics.

### Characteristics of subgroups “analgesic overuse (AO)” and “no analgesic overuse (noAO)”

Of 178 patients, 138 (78%) were overusing analgesics as defined by criterion B in the ICHD-II-Appendix classification [20]. In the subgroups “with” and “without” analgesic overuse mean age was 46 ± 12 years, and 42 ± 14 respectively, gender distribution was 51% and 50%, and mean duration of pain suffering was 7.8 ± 8.9 years and 8.0 ± 9.9 years, respectively. The percentage of patients with a history of migration was higher in the AO subgroup with 69%, as compared to noAO with 43% (*p* = 0.012), and to the overall population (63%). While 31% of patients with analgesic overuse reported a previous accident, this was similar to the incidence of in non-analgesic overusers (28%) and the overall sample population (30%). In patients with AO 59% had been diagnosed with anxiety and/or depression, in the noAO 20% only.

### Headache characteristics

**Figure 1:**
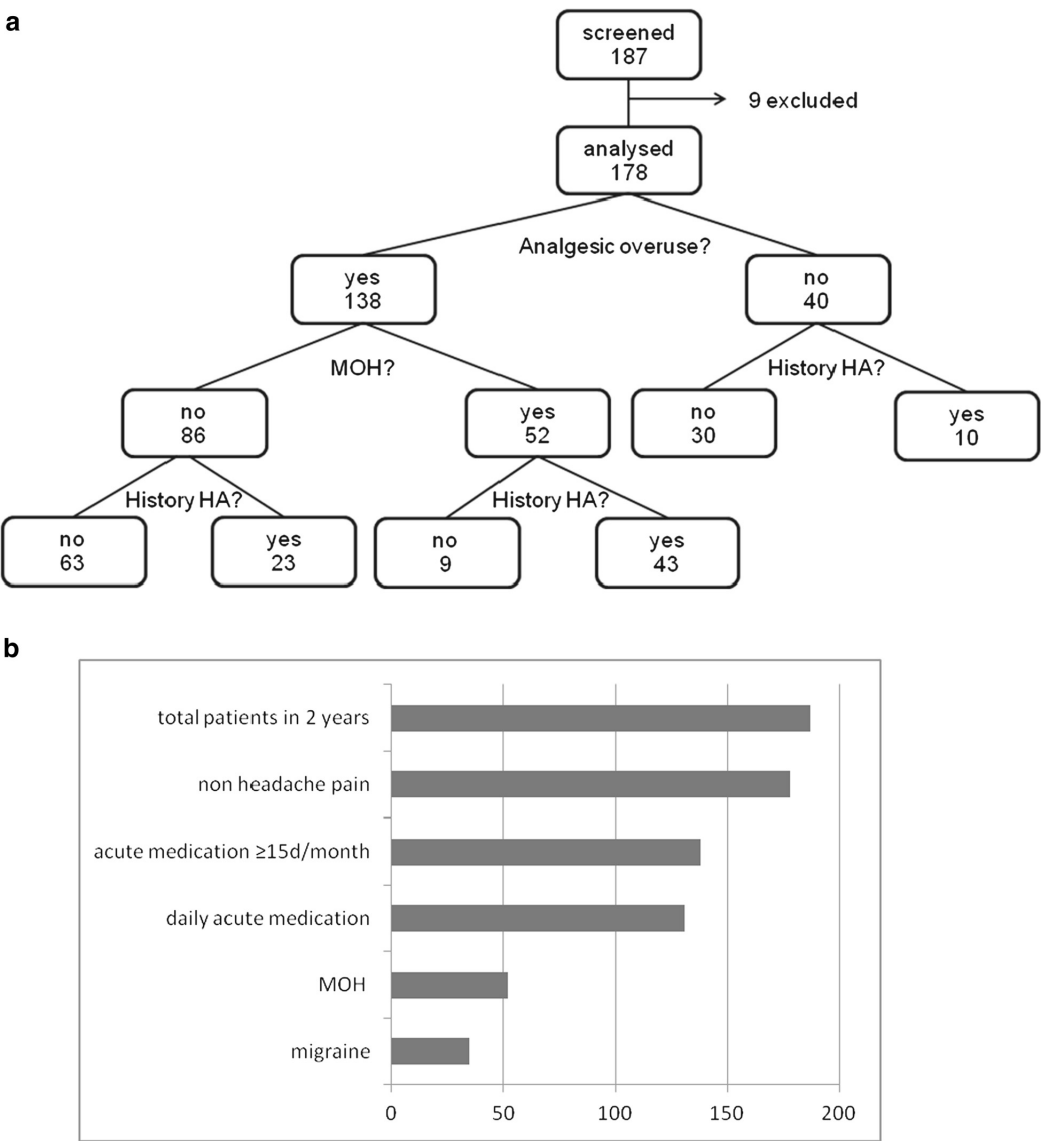
Patient population.

While 62 patients in the sample reported chronic headaches (CDH: headaches on 15 or more days per month), a total of 52 patients fullfilled the ICHD-II-Appendix criteria [20] for medication overuse headache (for an overview see Figure 1). This is 38% of the patients overusing analgesics (n = 138) and 29% of all patients with chronic pain in the sample (n = 178). There were 3 patients with CDH in the group with analgesic overuse who did not fullfill criterion C (see Table 1), and 7 patients who where not overusing analgesics. Therefore, the prevalence of CDH was 40% in the AO group (including MOH = 55/138) and 18% (=7/40) in the noAO group. The proportions of patients with a history of migraine and/or other primary headaches (i.e. tension type headache) were for MOH 67%, for AO only (without MOH) 27%, and for noAO 25% (see Figure 1). The prevalence of migraine was 67% (= 35/52) in the MOH patients, 23% (= 20/86) in the AO only group, and 20% (= 8/40) in the noAO group. The probability (OR) was 13.1 (CI [5.52; 31.01]) times higher for patients overusing analagesics to develop MOH when there was a history of primary headaches (see Table 3), and 6.8 times higher when they suffered from migraine.

**Table 3:** MOH and history of primary headache or migraine

**a) OR 13**.**1, CI [5.52; 31.01]**				
		**History of primary HA**		
		yes	no	
** MOH**	yes	43	9	52
	no	23	63	86
		63	75	138
**b) OR 6.8, CI [6.08; 28.14]**				
		**Migraine**		
		yes	no	
** MOH**	yes	35	17	52
	no	20	66	86
		55	83	138

### Associated factors for pain medication overuse

History of previous headache, history of migration, and comorbid depression were identified as significantly associated in the univariate analysis. The multiple logistic regression resulted in odds ratios of 2.5 (CI [1.13;5.44]) for a history of primary headache, 2.9 (CI [1.31;6.32]) for a history of migration, and 3.5 (CI [1.46;8.52]) for comorbid depression (see Table 4).

**Table 4:** Associated factors with chronification of headache

	**p-value**	**p-value after Bonferroni correction**	**Odds ratio [95% CI]**	**Odds ratio after multipl. log. regression**
**Age** Age + 10 years	0.09	0.703	–	
**Sex male**/ **female**	0.86	1.000	–	
**Duration of pain disorder**	0.76	1.000	–	
**History of migration**	0.0015	0.012	3.29 [1.51,7.36]	2.89 [1.13,5.44]
**History of headache**	0.0016	0.013	3.31 [1.51,7.46]	2.48 [1.31,6.32]
**Accident**	0.70	1.000	–	
**Depression**	0.0002	0.002	4.51 [1.85,12.25]	3.52 [1.46,8.52]
**Anxiety**	0.79	1.000	–	

Table 4 shows results of the univariate analysis of different factors. P-values and p-values after bonferroni correction are listed. Odds ratios and the 95% confidence interval are calculated only when p-values were significant. Also, odds ratios after multiple log. Regression are listed for age + 10 years, migration, history of headache or depression.

## Discussion

So far, five studies on MOH in chronic pain patients have been published [24-28]. Lance et al. found that 24 of 89 patients in a rheumatology clinic had headache on 4 to 28 days per month while taking more than 14 tablets per week; no significant difference in headache frequency between those taking more or less than 14 tablets per week was found [26]. Bowdler et al. reported 6 patients complaining of chronic headache out of 1411 consecutive cases (0.4%) in an anesthesiological pain clinic, of which 140 (9.9%) had regular non-opioid analgesic intake [25]. In 28 patients taking opioids regularly for bowel motility control after total colectomy, Wilkinson et al. found 8 patients with daily intake of opioids; while two of them suffered from chronic daily headache and both reported a history of previous migraine [27]. Bahra studied a population of 111 patients in a rheumatology clinic, identifying 103 patients with regular analgesic use; of those, 8 (7.6%), all migraineurs, suffered from CDH [24]. Finally, another population from a rheumatolgy clinic was studied by Williams et al., finding that 14 of 114 (12%) patients suffered from chronic daily headache, whereas 9 (8%) patients met the criteria for problable MOH according to ICHD-II revised criteria [28,30]. However, all those studies have been using diagnostic criteria other than current ICHD-II-Appendix definition for MOH, and therefore comparison with our data is difficult.

The prevalence of 29% for MOH in our pain population is higher than previously reported. Different reasons may explain our finding. First, ICHD-II-Appendix criteria enable more patients to be diagnosed with MOH at the time of the examination. The rationale behind this is to increase awareness for the problem and to allow more patients to benefit from specific treatment [20]. Reports of underestimation of MOH in clinical practice support these arguments [3,24]. Due to the retrospective nature of our study, the prevalence of MOH might have been overestimated because of incomplete information on the timing of headache chronification and beginning of medication overuse (criterion C of the definition, [20]). Also, our population consisted of patients referred to a tertiary pain clinic because of long-standing and complex pain problems, as reflected by the high number of cummulative diagnostic categories in the majority of cases. In Switzerland, healthcare system allows free access to specialists for most people. Patients may consult more than one specialist for their complex health problem, leading to prescription of analgesics by different doctors without knowledge of the patients current drug treatment. Many of the analgesics taken are even over the counter (OTC), what represents another risk factor for uncontrolled analgesic intake. Due to the retrospective nature of our study we have to take into account methodological limitations. These limitations apply to adequacy and bias of data collection due to quality of recorded information and inadequacy of patient’s reports, as already mentioned. However, more inclusive criteria would produce more false positive cases, i.e. patients with headache chronification unrelated to medication overuse, will be diagnosed with MOH [31]. Hence, some researchers have challenged the hypothesis of medication overuse leading to chronic hedache. They criticized that the primary clinical observation for the concept of MOH, i.e. improvement of headache after discontinuation of medication overuse, has never been demonstrated in a placebo-controlled trial, and has yet to be formally proved [32]. On the other hand, the fact that a subset of patients does improve in their headache pattern after withdrawal cannot be disregarded. The conclusion that we *can* draw from this controversy is that in some headache patients the headache will chronify as a consequence of the medication overuse, while in others the chronification is caused by other factors.

Comorbid depression, history of migration, history of headache and have been identified as independently associated factors for analgesic overuse in general, this is in line with previous studies [33-35]. The association of having a history of primary headaches among the MOH patients, is in line with other studies. This accounts especially for migraine. In contrast to Radat et al., we found no significant correlation between anxiety and medication overuse [36]. However, psychiatric evaluation was not done using standardized questionnaires.

## Conclusions

In summary, our study showed that MOH might be more prevalent in chronic pain patients than previously suggested. ICHD-II-Appendix criteria proved to be a good screening instrument to detect MOH, leading instantly to the first and most important therapeutic step of drug withdrawal. Our data also suggest that headache chronification may be associated with other factors than medication overuse. Finally, chronic pain patients are a population at risk for developping analgesic overuse; therefore, careful recording of drug intake is mandatory in these patients, especially in the ones with a history of migration, a history of headache and/or depression. There is no simple answer as to how to treat those patients correctly. Whether to stay i.e. untreated on low back pain instead of developping chronic headaches is an issue, which has to be discussed in depth with the individual patient at risk, and a clear aim of the treatment should be defined in any case.

## Competing interests

Possible conflicts of interest (including financial and other relationships) for each author include the following: *CWS*: no. *KM*: Received travel support for consulting or lecturing from the following companies: Pfizer AG, Zurich, Switzerland; Bristol-Myers Squibb SA, Baar, Switzerland; Mundipharma Medical Company, Basel, Switzerland; Janssen-Cilag AG, Baar, Switzerland; UCB, Bulle, Switzerland; Medtronic, Bern, Switzerland; Boston Scientific AG, Solothurn, Switzerland; B. Braun Medical AG, Sempach, Switzerland; Grünenthal Pharma Schweiz, Mitlödi; Switzerland; St. Jude Medical AG, Zurich, Switzerland. *DMS*: no. *EA*:Received honorarium and travel support for consulting or lecturing from the following companies: Grünenthal GmbH, Aachen, Germany and Mitlödi, Switzerland, Pfizer AG, Zürich, Switzerland. *DRS*: His academic department is receiving grant support from the Swiss National Science Foundation, Berne, Switzerland (grant numbers: 33CM30_124117 and 406440-131268), the Swiss Society of Anesthesiology and Reanimation (SGAR), Berne, Switzerland (no grant numbers are attributed), the Swiss Foundation for Anesthesia Research, Zurich, Switzerland (no grant numbers are attributed), Bundesprogramm Chancengleichheit, Berne, Switzerland (no grant numbers are attributed), CSL Behring, Berne, Switzerland (no grant numbers are attributed), Vifor SA, Villars-sur-Glâne, Switzerland (no grant numbers are attributed). DRS was the chairman of the ABC Faculty and is a member of the ABC Trauma Faculty which both are managed by Thomson Physicians World GmbH, Mannheim, Germany and sponsored by an unrestricted educational grant from Novo Nordisk A/S, Bagsvärd, Denmark and CSL Behring GmbH, Hattersheim am Main, Germany. In the past 5 years, DRS has received honoraria or travel support for consulting or lecturing from the following companies: Abbott AG, Baar, Switzerland, AMGEN GmbH, Munich, Germany, AstraZeneca AG, Zug, Switzerland, Bayer (Schweiz) AG, Zürich, Switzerland, Baxter S.p.A., Roma, Italy, B. Braun Melsungen AG, Melsungen, Germany, Boehringer Ingelheim (Schweiz) GmbH, Basel, Switzerland, Bristol-Myers-Squibb, Rueil-Malmaison Cedex, France and Baar, Switzerland, CSL Behring GmbH, Hattersheim am Main, Germany and Bern, Switzerland, Curacyte AG, Munich, Germany, Ethicon Biosurgery, Sommerville, New Jersey, USA, Fresenius SE, Bad Homburg v.d.H., Germany, Galenica AG, Bern, Switzerland (including Vifor SA, Villars-sur-Glâne, Switzerland), GlaxoSmithKline GmbH & Co. KG, Hamburg, Germany, Janssen-Cilag AG, Baar, Switzerland, Janssen-Cilag EMEA, Beerse, Belgium, Merck Sharp & Dohme-Chibret AG, Opfikon-Glattbrugg, Switzerland, Novo Nordisk A/S, Bagsvärd, Denmark, Octapharma AG, Lachen, Switzerland, Organon AG, Pfäffikon/SZ, Switzerland, Oxygen Biotherapeutics, Costa Mesa, CA, Pentapharm GmbH (now tem Innovations GmbH), Munich, Germany, ratiopharm Arzneimittel Vertriebs-GmbH, Vienna, Austria, Roche Pharma (Schweiz) AG, Reinach, Switzerland, Schering-Plough International, Inc., Kenilworth, New Jersey, USA, Vifor Pharma Deutschland GmbH, Munich, Germany, Vifor Pharma Österreich GmbH, Vienna, Austria, Vifor (International) AG, St. Gallen, Switzerland. *ARG*: Received honoraria or travel support for consulting or lecturing from the following companies: Allergan, Astra Zeneca, Eli Lilly, Merck Sharp & Dohme-Chibret AG, Pfizer, Sandoz AG, furthermore he received academic research grants from Allergan and Almirall. *PS*: Research Support: Swiss National Foundation; Janssen Cilag; Selo Foundation; „Erwin Schrödinger-Stipendium“, Research Funds of RehaClinic Bad Zurzach and the Cantonal Hospital Baden; Employee of RehaClinic Bad Zurzach; Consultant – Advisory Boards of Pfizer, Allergan; Speaker‘s Bureau – Allergan, Almirall, Pfizer.

## Authors’ contributions

Conception and design: PS, CWS, KM, EA. Analysis and interpretation of the data: CWS, KM, ARG, PS, DMS, DRS, EA. Drafting of the article: CWS, KM, ARG, PS, DMS. Critical revision of the article for important intellectual content: PS, EA, DRS. Collection and assembly of data: CWS, PS. Statistical expertise: KR, KM, ARG. All authors read and approved the final manuscript.
